# Diagnostic and prognostic performance of PATHFAST Presepsin in patients with SIRS and early sepsis

**DOI:** 10.1186/cc14143

**Published:** 2015-03-16

**Authors:** E Spanuth, R Carpio, R Thomae

**Affiliations:** 1 DIAneering GmbH, Heidelberg, Germany; 2 Hospital Nacional Edgardo Rebagliati Martins-EsSalud, Lima, Peru; 3 Mitsubishi Chemical Europe, Düsseldorf, Germany

## Introduction

Presepsin (sCD14-ST) serves as a mediator of the response to infectious agents. First evidence suggested that presepsin may be utilized as a sepsis marker.

## Methods

Presepsin was determined at presentation (T0), after 8, 24 and 72 hours in 123 individuals admitted with signs of SIRS and/ or infection. Primary endpoint was death within 30 days. Presepsin was determined using the POC assay PATHFAST Presepsin (Mitsubishi Chemical, Japan).

## Results

Mean presepsin concentrations of the patient group at presentation and of the control group were 1,945 and 130 pg/ml, respectively (*P *< 0.0001). Baseline presepsin differed highly significant between patients with SIRS, sepsis, severe sepsis and septic shock. Twenty-four patients died during 30 days. The 30-day mortality was 19.5% in total, ranging from 10 to 32% between the first and the fourth quartile of presepsin concentration. Nonsurvivors showed high presepsin values with increasing tendency during the course of the disease while in surviving patients this tendency was decreasing. See Table 1.

**Table 1:** Presepsin levels during the course of the disease in survivors and nonsurvivors

PSEP, median (IQR) (pg/ml)				
	**T0**	**T8 hours**	**T24 hours**	**T72 hours**

Survivors	590 (345 to 1,396)	622 (367 to 1,912)	574 (336 to 1,610)	533 (324 to 1,246)
Nonsurvivors	1,763 (705 to 6,616)	1,859 (1,001 to 5,744)	1,731 (809 to 4,586)	2,056 (811 to 5,540)
*P *value	0.0046	0.0005	0.0003	0.0013

## Conclusion

Presepsin demonstrated a strong relationship with disease severity and outcome. Presepsin provided reliable discrimination between SIRS and sepsis as well as prognosis and early prediction of 30day mortality already at admission. Presepsin showed close association with the course of the disease.

